# Localized lymphoid hyperplasia with prominent IgG4-positive plasma cells in the inferior turbinate mimicking IgG4-related disease

**DOI:** 10.3389/fimmu.2026.1898657

**Published:** 2026-10-14

**Authors:** Doo Hee Han, Ji In Jung, Ju Yeon Kim, Jiwon Koh, Jun Won Park, Eun Bong Lee, Jin Kyun Park

**Affiliations:** 1 Department of Otorhinolaryngology, Seoul National University College of Medicine, Seoul National University Hospital, Seoul, Republic of Korea; 2 Division of Rheumatology, Department of Internal Medicine, Seoul National University College of Medicine, Seoul, Republic of Korea; 3 Division of Rheumatology, Department of Internal Medicine, Chung-Ang University Gwangmyeong Hospital, Gwangmyeong-si, Republic of Korea; 4 Department of Pathology, Seoul National University College of Medicine, Seoul National University Hospital, Seoul, Republic of Korea

**Keywords:** hyperplasia, immunoglobulin G4-related disease, lymphoid tissue, mass, nasal cavity

## Abstract

**Objectives:**

Localized lymphoid masses of the inferior turbinate with prominent IgG4-positive plasma cell infiltration are occasionally encountered in clinical practice, yet their nature and relationship to IgG4-related disease (IgG4-RD) remain uncertain. This study aimed to characterize the clinical, histopathological, and therapeutic features of these lesions.

**Methods:**

We retrospectively reviewed patients presenting with localized submucosal masses of the inferior turbinate identified on nasal endoscopy at Seoul National University Hospital between January 2020 and May 2023. Biopsies were performed in cases with mucosal disruption, including crusting or bleeding. Classification of IgG4-RD was based on the 2019 American College of Rheumatology/European League Against Rheumatism criteria. The 2011 Comprehensive diagnosis criteria were applied to the patients with available biopsies.

**Results:**

Of 14,103 patients undergoing nasal endoscopy, 41 (0.3%) demonstrated localized inferior turbinate submucosal lesions. Eighteen patients underwent biopsy, all of which revealed dense lymphoplasmacytic infiltration with variable IgG4-positive plasma cell counts. An IgG4+/IgG+ plasma cell ratio exceeding 40% was observed in 10 cases (55.6%). However, characteristic histopathological features of IgG4-RD, including storiform fibrosis and obliterative phlebitis, were absent. Only two patients fulfilled the 2019 classification criteria for IgG4-RD, both of whom had extra-nasal involvement. According to the 2011 Comprehensive diagnosis criteria, 2 patients were classified as definite IgG4-RD. Spontaneous improvement during observation occurred in a minority of patients. Surgical treatment resulted in durable symptom control with minimal recurrence, whereas corticosteroid therapy showed variable effectiveness.

**Conclusions:**

Localized IgG4-rich lymphoid hyperplasia of the inferior turbinate may represent a distinct benign inflammatory lesion, mimicking a sinonasal manifestation of IgG4-RD. Further studies are required to clarify its pathophysiology, optimal management, and long-term outcomes.

## 1 Introduction

IgG4-related disease (IgG4-RD) is a rare, multi-system fibro-inflammatory condition characterized by dense lymphoplasmacytic infiltration, storiform fibrosis, obliterative phlebitis, tissue infiltration by IgG4-positive plasma cells, and frequently elevated serum IgG4 concentrations (1–3). In the head and neck region, IgG4-RD most commonly affects the lacrimal, orbital, parotid, and submandibular glands, as well as the thyroid gland and cervical lymph nodes. In contrast, sinonasal involvement is uncommon (4). When present, sinonasal IgG4-RD typically manifests with nasal obstruction, chronic rhinosinusitis, or epistaxis (5, 6) and rarely presents as hypertrophy or sclerosis of the inferior turbinate (7).

Localized masses of the inferior turbinate may cause nasal symptoms but differ from diffuse turbinate hypertrophy. These lesions are typically focal, confined to the anterior portion of the inferior turbinate, and may occur independently of rhinitis or sinusitis. Histopathological examination often demonstrates dense lymphoplasmacytic infiltration with IgG4-positive plasma cells, mimicking the features of IgG4-RD (6). However, it remains unclear whether these lesions represent a localized nasal manifestation of systemic IgG4-RD or a distinct, previously unclassified inflammatory disease entity.

Distinguishing IgG4-RD from benign lymphoid hyperplasia has important clinical implications. IgG4-RD generally requires prolonged systemic glucocorticoid therapy, which carries substantial risks including diabetes, osteoporosis, cardiovascular disease, and cataracts (8–10). In contrast, benign lymphoid lesions may be managed conservatively or treated surgically if symptomatic. This study aimed to characterize the clinical, histopathological, and therapeutic features of localized lymphoid masses of the inferior turbinate and to clarify their relationship to IgG4-RD.

## 2 Methods

### 2.1 Study design and patients

This retrospective cohort study included patients who underwent nasal endoscopic evaluation for various nasal symptoms at Seoul National University Hospital between January 2020 and May 2023. Biopsies were performed when inferior turbinate masses demonstrated mucosal disruption, including crusting or bleeding. Patients subsequently underwent systemic evaluation for autoimmune or inflammatory disease (**Figure 1**). Patients were treated at the discretion of the primary physician.

**Figure 1:**
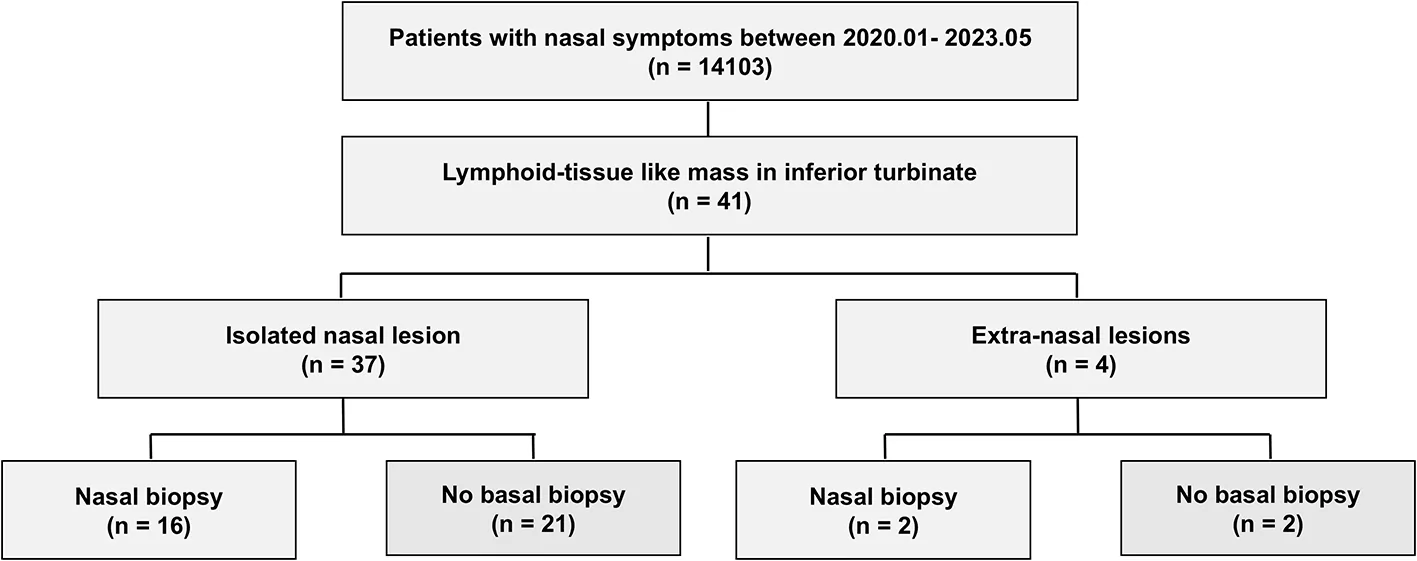
Overview of patient flow.

The study was conducted in accordance with the revised Declaration of Helsinki and was approved by the Institutional Review Board of Seoul National University Hospital (IRB No. 2212-139-1389). Informed consent was waived due to the retrospective study design.

### 2.2 Endoscopic examination

All patients underwent standard nasal endoscopic examination conducted by a rhinologist (HDH) using 3 mm 0-degree rigid endoscopes (Karl Storz, Tuttlingen, Germany) in an outpatient clinic setting.

### 2.3 Histopathological evaluation and immunohistochemistry

Biopsy specimens obtained during endoscopy were fixed in 10% neutral buffered formalin for 24–72 hours, processed, and subsequently embedded into paraffin blocks. From these paraffin blocks, 4-μm sections were cut for hematoxylin-eosin (H&E) staining and immunohistochemistry (IHC). The immunohistochemistry analysis was performed using anti-IgG rabbit polyclonal antibody (A0423; Dako, Glostrup, Denmark) and anti-IgG4 mouse monoclonal antibody (clone MRQ-44; Ventana Medical Systems, Tucson, AZ, USA) with the BenchMark XT System (Ventana Medical System), following the manufacturer’s instructions. Tonsil tissue was used as a positive control.

### 2.4 Evaluation of IgG4-RD

All subjects underwent a comprehensive medical assessment, encompassing evaluations of skin, joint, and soft tissue involvement. Laboratory analysis included erythrocyte sedimentation rate (ESR), and high-sensitivity C-reactive protein (hs-CRP). Autoantibodies such as rheumatoid factor (RF), antinuclear antibody (ANA), and serum IgG4 levels were examined in cases where an autoimmune disease was clinically suspected.

IgG4-RD was diagnosed according to the 2019 American College of Rheumatology/European League Against Rheumatism classification criteria (ACR/EULAR) of IgG4-RD (11). Histologic IgG4-RD was defined by the presence of two or more of the following three features: dense lymphocytic infiltrates, storiform fibrosis, and phlebitis. Immunohistochemical IgG4-RD was defined by the presence of over 10 IgG4+ cells per high power field (HPF) and the IgG4/IgG ratio exceeding 0.4 (3). In patients with available biopsy specimens, the 2011 comprehensive diagnostic criteria for IgG4-RD were also applied (12). These criteria integrate characteristic organ swelling or mass-forming lesions, serum IgG4 levels, and histopathologic findings to categorize patients as having definite, probable, possible, or no IgG4-RD.

### 2.5 Statistical analysis

Continuous variables were presented as means ± standard deviations or medians with interquartile ranges (IQR), as appropriate. Categorical variables were expressed as absolute counts and percentages. Group comparisons were performed using the Student’s *t*-test or Mann–Whitney *U* test for comparisons between two groups and one-way ANOVA or the Kruskal-Wallis test for comparisons among three or more groups, as appropriate. Categorical variables were compared using the chi-square test or Fisher’s exact test, as appropriate. Statistical significance was defined at p < 0.05. R-Studio was utilized for all analyses.

## 3 Results

### 3.1 Patient characteristics

Among the 14,103 patients evaluated for nasal symptoms between January 2020 and May 2023 at the ENT clinic of the Seoul National University Hospital, 41 (0.3%) patients exhibited a submucosal lymphoid hyperplasia in the inferior turbinate during rhinoscopy (**Figure 1**). Thirty-seven patients had isolated nasal disease, while four exhibited extra-nasal involvement. Biopsies were performed on 16 out of the 37 patients with isolated nasal lesions and two out of the four patients with extra-nasal involvement, all of whom exhibited features of mucosal damage, including crusting and bleeding.

The mean age of the 41 patients was 51.5 ± 16.4 years, with a male predominance (75.6%). The median symptom duration was 36.0 [4.1- 105.0] months. Rhinorrhea was the most common symptom, observed in 31.7% of patients, followed by nasal obstruction (29.3%) and nasal mass (12.2%). Notably, 15 (36.6%) of 41 patients reported allergies. Patients with extra-nasal symptoms had higher total IgG and IgG4 levels than those with isolated nasal lesions (**Table 1**).

**Table 1:** Baseline characteristics of study participants.

Characteristics	Isolated nasal lesions (N = 37)	Extra-nasal involvement (n = 4)	*P* Value
Age, years	51.9 ± 15.7	47.8 ± 25.2	0.634
Sex, male	29 (78.4)	2 (50.0)	0.520
Duration, months	27.2 [3.1 - 83.2]	105.3 [47.3 - 183.8]	0.195
Nasal symptoms	NA	NA	NA
Rhinorrhea	13 (35.1)	0 (0)	0.385
Nasal obstruction	10 (27.0)	2 (50.0)	0.703
Nasal mass	5 (13.5)	0 (0)	1.000
Sneezing	5 (13.5)	0 (0)	1.000
Postnasal drip	3 (8.1)	0 (0)	1.000
Nasal pain	3 (8.1)	0 (0)	1.000
Crusting	6 (16.2)	0 (0)	0.899
Nasal bleeding	2 (5.4)	0 (0)	1.000
Allergy	11 (29.7)	4 (100)	**0.026**
Laboratory tests			
WBC, x 10^3^/mm^3^	5730.0 [4865.0 - 6910.0]	9500.0 [6660.0 - 10870.0]	0.087
Eosinophil, 10^3^/mm^3^	2.5 [1.9 - 4.0]	2.7 [1.6 - 4.2]	0.957
Hemoglobin, g/dL	13.3 ± 1.2	14.0 ± 0.8	0.229
Platelet, 1000/mm^3^	230.0 [216.0 - 265.5]	275.0 [248.5 - 291.5]	0.137
ESR, mm/hr	12.0 [7.0 - 20.0]	13.5 [8.0 - 19.5]	0.953
CRP, mg/dL	0.1 [0.0 - 0.2]	0.1 [0.1 - 0.2]	1.000
Serum IgG, mg/dL	1251.5 ± 122.7	1838.0 ± 275.8	**0.038**
Serum IgG4, mg/dL	55.2 ± 48.4	206.9 ± 111.8	**0.024**

Data are presented as the mean ± standard deviation, median [IQR] or number (%). Numbers in bold indicate a statistical significance.

ESR, erythrocyte sedimentation rate; CRP, C-reactive protein; WBC, White blood cells.

### 3.2 Endoscopic findings

Rhinoscopy revealed yellowish sessile submucosal masses with intact overlying mucosa in the anterior portion of the inferior turbinate (**Figures 2A–C**). In 18 patients, the overlying mucosa showed signs of hemorrhage and crusting, and these lesions were biopsied.

**Figure 2:**
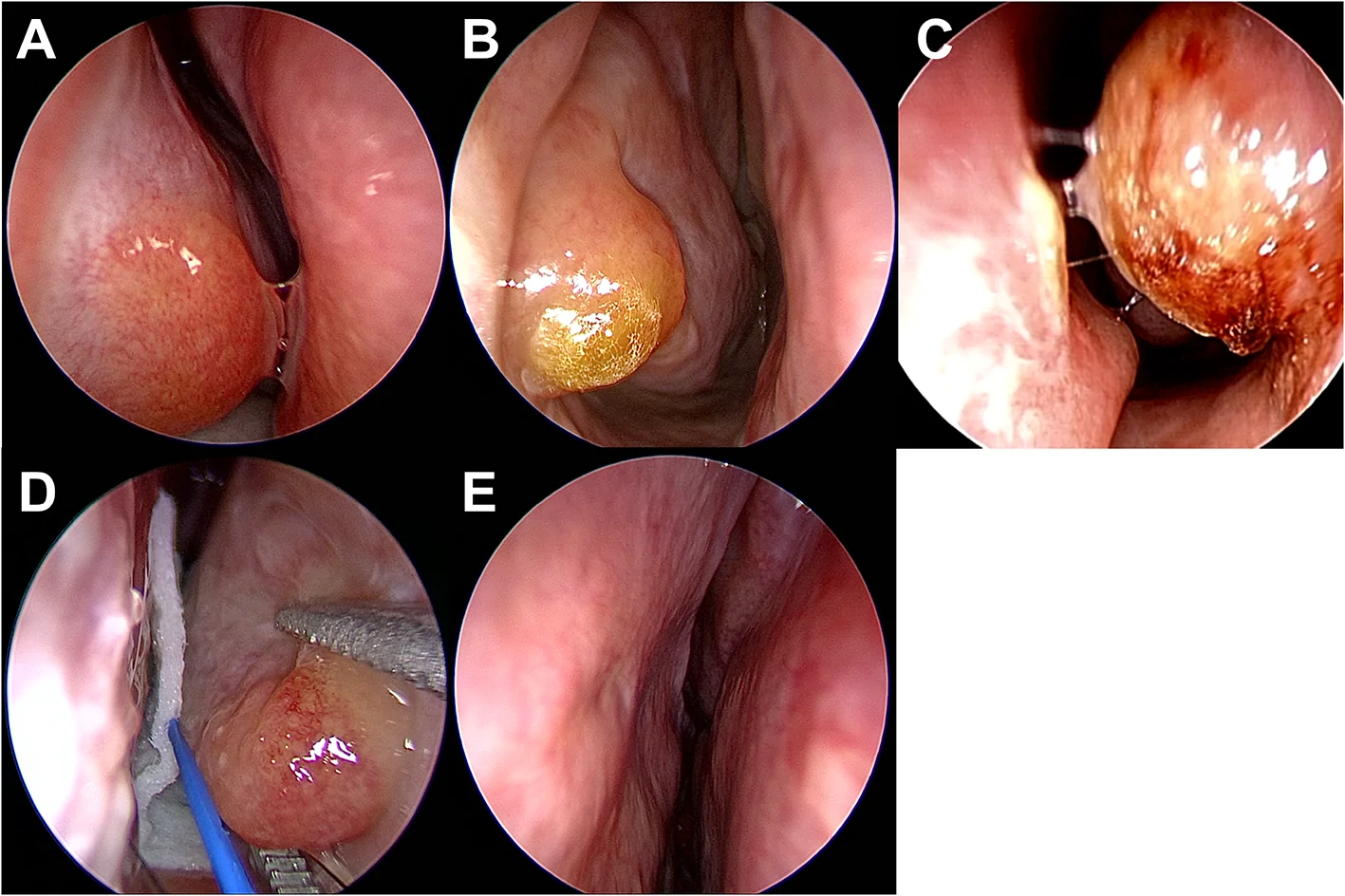
Representative endoscopic findings of lymphoid nasal mass. Rhinoscopy reveals sessile submucosal masses with intact overlying mucosa in the anterior portion of the inferior turbinate. Representative images of a mild **(A)**, moderate **(B)** and a severe case with bloody crust **(C)** were depicted. **(D)** The lesion was surgically removed. **(E)** The lesion did not recur three months after surgical resection.

### 3.3 Histologic characteristics

All 18 biopsies exhibited dense lymphoplasmacytic infiltrates. Lymphoid follicle formation was identified in eight (44.4%) cases. Fibrosis was observed in 10 (55.6%) cases but none of them exhibited storiform fibrosis. Obliterative phlebitis was absent (**Table 2**). Immunohistochemical analysis revealed the substantial presence of CD3-positive T cells and CD20-positive B cells within the infiltrates, along with a significant amount of IgG and IgG4 positive cells. Ki-67 expression co-localized with CD20-positive B cells (**Figure 3**). Furthermore, all samples demonstrated varying amounts of IgG4-expressing plasma cells with ten (55.6%) cases exhibiting the IgG4+/IgG+ ratio exceeding 40%, thereby meeting the criteria for the immunohistologic diagnosis of IgG4-RD (**Table 2**).

**Table 2:** Histological findings of nasal biopsy.

Biopsy finding	Total (n = 18)
Lymphoid follicle	8 (44.4%)
Fibrosis	10 (55.6%)
Storiform fibrosis	0 (0)
Phlebitis	0 (0)
IgG, cells/HPF	353.0 ± 230.1
IgG4, cells/HPF	80.0 [40.0 - 102.0]
IgG4/IgG ratio, %	40.0 [9.6 - 48.0]
immunohistochemical diagnosis of IgG4-RD	10 (55.6%)

Data are presented as the mean ± standard deviation, median [IQR] or number (%).

HPF, high power field; Ig, immunoglobulin; IgG4-RD, IgG4-related disease.

**Figure 3:**
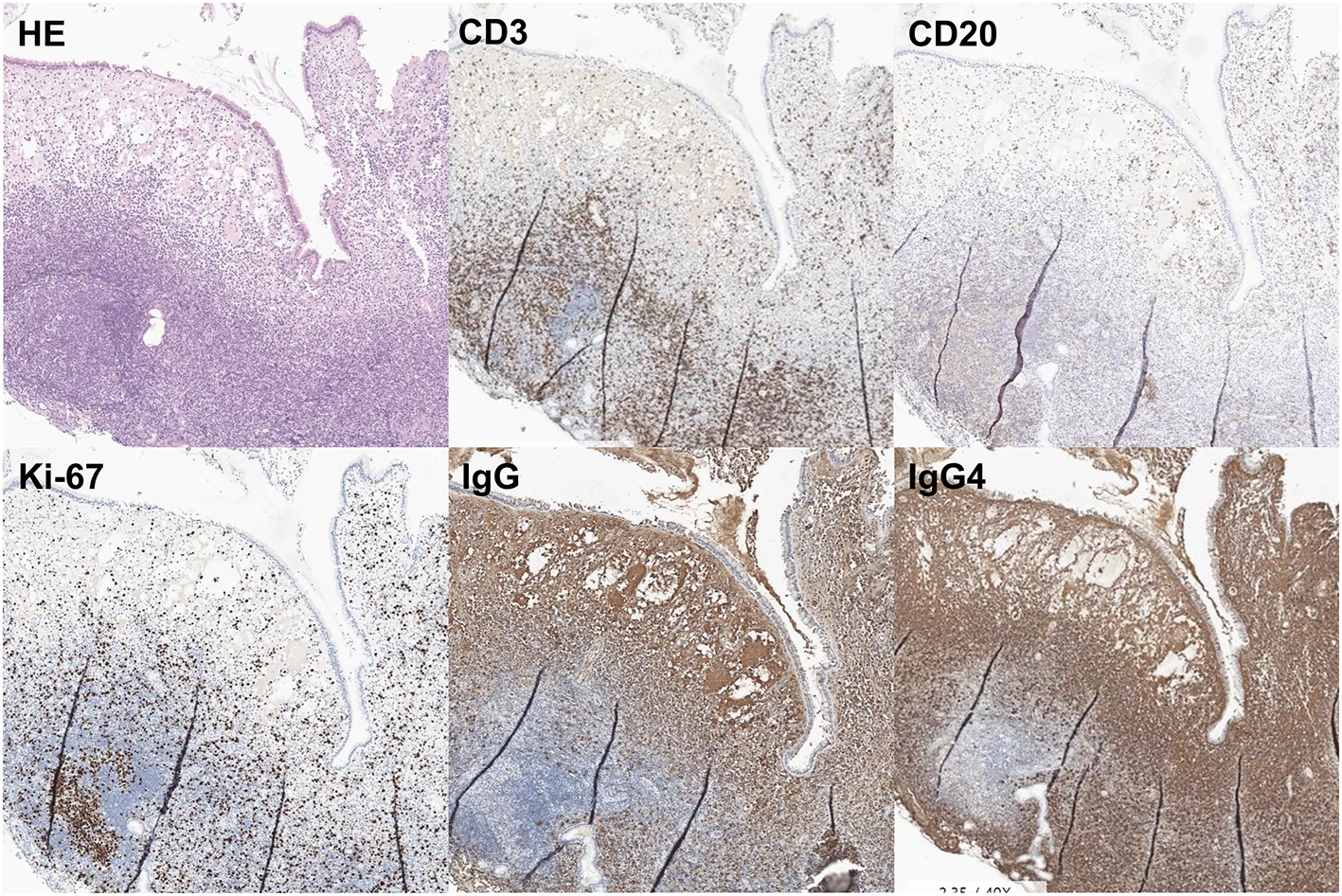
Histopathological examination of nasal masses. Hematoxylin and eosin (H&E) staining revealed a dense lymphoplasmacytic infiltrate, forming a prominent lymphoid structure. Abundant CD3-positive T cells and CD20-positive B cells were observed, along with substantial IgG and IgG4 production. Notably, more than 100 IgG4-positive plasma cells were present per high-power field, with an IgG4/IgG plasma cell ratio exceeding 40%.

### 3.4 Diagnosis according to the 2019 ACR/EULAR classification criteria and the 2011 comprehensive diagnosis criteria

All patients met the entry criteria based on the lymphoid mass, while none met the exclusion criteria of the 2019 ACR/EULAR classification criteria for IgG4-related disease. Among the 41 patients, only two were classified as having IgG4-RD according to the 2019 ACR/EULAR classification criteria. Both patients had extra-nasal involvements. The first case, a 27-year-old man, displayed a constellation of chronic pansinusitis, pancreatitis, and parotid enlargement. With a serum IgG4 level of 164.7 mg/dL (normal upper limit: 157 mg/dL), his total IgG4-RD score was 33. The second patient was a 76-year-old man who presented with pansinusitis, lacrimal gland engagement, pituitary gland lesion, and periaortic infiltrate. His serum IgG4 level was 68.2 mg/dL, and the IgG4-EULAR/ACR score was 28. Nasal biopsies from two IgG4-RD patients displayed numerous IgG-positive cells. However, lymphoid follicle formation, storiform fibrosis, and phlebitis were absent in both patients.

The 2011 Comprehensive diagnosis criteria were applied to the 18 patients with available biopsies. Of these, 9, 7, and 2 patients were classified as possible, probable, and definite IgG4-RD, respectively.

### 3.5 Treatment and outcome

Patients were treated at the discretion of their physicians. Of 41 patients, 18 (43.9%) patients experiencing substantial nasal symptoms were treated; 10 underwent surgery, 4 received corticosteroid therapy, and 4 received both. Clinical and laboratory characteristics were generally comparable across treatment groups, except for the prevalence of crusting (**Table 3**). Surgical resection demonstrated a high success rate, achieving no recurrence in 9 (90%) of 10 patients whereas corticosteroid therapy was less effective, with only 2 (50%) of 4 patients showing a response (**Table 4**). Among the 4 patients who received both surgery and corticosteroid, only 2 (50%) patients improved. In contrast, 23 patients were managed with observation alone. During the follow-up, spontaneous improvement occurred in 5 patients (21.7%), whereas the remaining 18 (78.3%) had persistent but stable lesions without clinical progression during follow-up. None of the patients managed conservatively subsequently required surgical intervention or systemic corticosteroid therapy.

**Table 3:** Baseline characteristics of study participants by treatment.

Characteristics	Observation (n=23)	CS (n=4)	Surgery (n=10)	CS + Surgery (n=4)	*P* Value
Age, years	54.0 ± 15.6	53.8 ± 20.8	47.5 ± 18.3	45.0 ± 15.0	0.622
Sex, male	19 (82.6%)	2 (50.0%)	7 (70.0%)	3 (75.0%)	0.531
Duration, months	59.6 ± 71.0	116.7 ± 99.1	50.7 ± 77.8	99.6 ± 174.1	0.524
Follow-up, months	51.1 ± 7.1	52.5 ± 6.6	50.3 ± 7.2	62.5 ± 4.5	**0.027**
Nasal symptoms					
Rhinorrhea	11 (47.8%)	0 (0.0%)	2 (20.0%)	0 (0.0%)	0.069
Nasal obstruction	7 (30.4%)	1 (25.0%)	3 (30.0%)	1 (25.0%)	0.993
Nasal mass	1 (4.3%)	1 (25.0%)	1 (10.0%)	2 (50.0%)	0.062
Sneezing	5 (21.7%)	0 (0.0%)	0 (0.0%)	0 (0.0%)	0.216
Postnasal drip	3 (13.0%)	0 (0.0%)	0 (0.0%)	0 (0.0%)	0.469
Nasal pain	1 (4.3%)	0 (0.0%)	2 (20.0%)	0 (0.0%)	0.347
Crusting	1 (4.3%)	1 (25.0%)	1 (10.0%)	3 (75.0%)	**0.003**
Nasal bleeding	2 (8.7%)	0 (0.0%)	0 (0.0%)	0 (0.0%)	0.649
Allergy	9 (39.1%)	3 (75.0%)	1 (10.0%)	2 (50.0%)	0.113
Laboratory tests					
WBC, x 10^3^/mm^3^	6440.6 ± 3103.4	8627.5 ± 3109.5	5652.0 ± 1395.8	5825.0 ± 1078.6	0.280
Eosinophil, 10^3^/mm^3^	2.8 ± 2.0	3.4 ± 2.1	3.5 ± 2.2	3.1 ± 0.7	0.858
Hemoglobin, g/dL	13.1 ± 1.2	13.3 ± 2.0	13.6 ± 1.1	13.7 ± 0.8	0.640
Platelet, 1000/mm^3^	236.8 ± 50.9	257.2 ± 28.0	249.3 ± 56.8	256.0 ± 39.9	0.805
ESR, mm/hr	11.6 ± 6.7	26.2 ± 19.2	13.0 ± 9.0	16.0 ± 10.9	0.102
CRP, mg/dL	0.2 ± 0.3	0.2 ± 0.1	0.4 ± 0.6	0.1 ± 0.1	0.743
Serum IgG, mg/dL	1360.0	1626.3 ± 415.3	1270.5 ± 127.9	1193.3 ± 145.3	0.161
Serum IgG4, mg/dL	NA	138.7 ± 114.3	71.5 ± 57.6	99.1 ± 138.6	0.605

Data are presented as the mean ± standard deviation. Numbers in bold indicate a statistical significance. NA, not applicable; ESR, erythrocyte sedimentation rate; CRP, C-reactive protein; CS, corticosteroid; WBC, White blood cells.

**Table 4:** Summary of treatment and outcomes in 41 patients with localized lymphoid hyperplasia.

Treatment group	Improved
CS only (n = 4)	2 (50.0)
Surgery only (n = 10)	9 (90)
Surgery + CS (n =4)	2 (50.0)
Observation (n = 23)	5 (21.7)

Data are presented as number (%). CS, corticosteroids.

The two patients with IgG4-RD received only medical treatment. The 27-year-old patient with IgG4-RD responded well to corticosteroids and immunosuppressants, including rituximab. In the case of the 76-year-old patient with IgG4-RD, extra-nasal lesions responded to corticosteroids, while the nasal symptoms such as sinusitis did not respond.

## 4 Discussion

In this study, lymphoid masses located in the anterior portion of the inferior turbinate demonstrated dense lymphoplasmacytic infiltration with IgG4-producing cells, immunohistochemically resembling IgG4-related disease (IgG4-RD). Although lymphoid follicle formation was frequently observed, neither storiform fibrosis nor obliterative phlebitis was identified in these lesions. None of the patients fulfilled the 2019 ACR/EULAR classification criteria for IgG4-RD unless concomitant extra-nasal IgG4-RD involvement was present. Clinically, nasal symptoms improved following surgical resection but showed limited responsiveness to corticosteroid therapy. Collectively, these findings suggest that these nasal lymphoid lesions might represent a distinct benign chronic inflammatory disease entity rather than a manifestation of sinonasal IgG4-RD.

Nasal polyps and turbinate hypertrophy are common causes of nasal obstruction. Nasal polyps arise from chronic inflammation, often associated with asthma, recurrent infections, allergy, or drug hypersensitivity, resulting in painless mucosal overgrowth that is typically asymptomatic unless airflow is compromised. Similarly, turbinate hypertrophy results from chronic mucosal irritation, leading to diffuse enlargement driven by inflammation and angiogenesis (13). Sinonasal involvement in IgG4-RD is rare although the head and neck are the second most frequently affected region after the pancreas. IgG4-RD of the head and neck typically presents as Mikulicz’s disease, Küttner’s tumour, or Riedel’s thyroiditis (4, 6). Sinonasal IgG4-RD has been reported to involve bone and soft tissue, causing chronic sinusitis with bleeding and sclerosis (14). It is often associated with other systemic manifestations, including elevated serum IgG4 levels. Therefore, differentiating this potentially aggressive form of sinonasal IgG4-RD from benign chronic inflammatory conditions with IgG4-positive cells is clinically important.

In the present study, endoscopy revealed sessile submucosal masses with yellowish overlying mucosa, occasionally accompanied by bloody crusting. These lesions were focal and well localized, in contrast to turbinate hypertrophy, which typically involves diffuse enlargement of the entire turbinate with or without mucosal swelling. Their preferential localization to the anterior portion of the inferior turbinate suggests a possible reaction to environmental exposures, such as cold or dry airflow, fine particulate matter, or allergens, particularly given that approximately one-third of patients reported allergic disease.

Histologically, all patients demonstrated dense lymphoplasmacytic infiltration without overt mucosal or submucosal tissue proliferation, resulting in sessile mass formation. Abundant T cells, B cells, and IgG-producing plasma cells were observed. In half of the biopsy specimens, lymphoid follicles resembling tertiary lymphoid organs (TLOs) were identified. TLOs have been described in other chronic autoimmune diseases, including the synovial membrane in rheumatoid arthritis, where they are often associated with more active disease and poorer outcomes (15). Further research is required to determine whether TLO has pathological or prognostic significance. None of the biopsies exhibited storiform fibrosis or phlebitis, which are hallmark histologic features of IgG4-RD, although half of the patients met immunohistochemical criteria of IgG4-RD (i.e., a ratio of >40% of IgG4 to IgG+ cells) (3). Importantly, IgG4 expression is not specific to IgG4-RD, as class switching to IgG4 may occur in a variety of chronic inflammatory conditions, including granulomatosis with polyangiitis and allergic disease (16–20). Serum IgG4 concentrations were not elevated in patients with isolated nasal lymphoid masses, in contrast to other head and neck manifestations of IgG4-RD, such as salivary or lacrimal gland involvement, which are often associated with markedly elevated serum IgG4 levels (21, 22). Taken together, the absent storiform fibrosis, phlebitis and normal serum IgG4 levels suggest that nasal lymphoid masses are distinct from other IgG4-RD in head and neck. Accordingly, only 2 of 41 patients were diagnosed with IgG4-RD according to the 2019 ACR/EULAR criteria of IgG4-RD (11), and these 2 patients had extensive extra-nasal manifestations of IgG4-RD. However, normal serum IgG4 levels do not exclude IgG4-RD, and the 2019 ACR/EULAR criteria were designed primarily for research classification with high specificity rather than for clinical diagnosis. Their sensitivity may therefore be limited in patients with single-organ disease, particularly when serum IgG4 levels are normal and the involved site, such as the sinonasal region, is not recognized as a typical organ for entry into the classification algorithm.

An additional distinguishing feature was the differential treatment response. While patients responded favorably to surgical resection, corticosteroid therapy was largely ineffective. IgG4-RD is characteristically highly responsive to corticosteroids, and steroid unresponsiveness is incorporated as an exclusion criterion in the ACR/EULAR classification system (11). The limited response to corticosteroids observed in this cohort therefore further argues that these lesions might not represent a nasal manifestation of IgG4-RD. In addition, the clinical course of untreated lesions was generally indolent and non-progressive. Among the 23 patients managed with observation, 5 (21.7%) showed spontaneous improvement, while the remaining 18 (78.3%) had persistent but stable lesions without clinical progression during follow-up. None subsequently required surgical intervention or systemic corticosteroid therapy. These findings suggest that, although spontaneous resolution is uncommon, localized inferior turbinate lesions may remain stable without treatment in many patients. However, given the retrospective nature of the study and limited follow-up, further longitudinal studies are needed to define their long-term natural history and determine the optimal management strategy.

Defining this newly recognized disease entity has clinical implications. Despite its immunohistological resemblance to IgG4-RD, this condition appears to be localized and benign. Mildly symptomatic lesions may be managed conservatively with close observation, whereas more symptomatic cases can be effectively treated surgically with an excellent prognosis. Systemic glucocorticoids may be appropriate in patients with extra-nasal IgG4-RD, while their use for localized nasal lesions should be individualized according to disease severity, clinical response, and recurrence. Given the rarity of this condition, identified in only 41 patients (0.3%) among 14,103 individuals undergoing nasal endoscopy, the true population incidence is likely to be even lower. Nevertheless, awareness of this entity is essential, and it should be considered in the differential diagnosis of focal inferior turbinate lesions.

As a retrospective study, this study has several limitations. First, there is potential for substantial selection bias and confounding by indication. Biopsy was not performed in all patients with localized inferior turbinate lesions. Biopsy was generally reserved for lesions with concerning mucosal changes, such as crusting or bleeding. Consequently, the histopathologic findings were derived from a selected subset of patients and may not represent the full spectrum of localized inferior turbinate lesions. Serologic testing was not performed systematically in all patients but rather at the discretion of the treating physician when clinically indicated, reflecting routine clinical practice. Therefore, incomplete serologic data may have limited our ability to fully characterize the systemic immunologic features of the study population. Similarly, treatment was determined at the discretion of the treating physician rather than according to a standardized protocol, resulting in potential confounding by indication. Although no significant differences in baseline clinical characteristics were observed among the treatment groups, residual confounding related to treatment selection cannot be excluded. Therefore, comparisons of treatment responses in this retrospective, non-randomized cohort should be interpreted with caution.

Second, the study included only patients with polypoid lesions of the inferior turbinate and therefore may not capture the broader spectrum of sinonasal IgG4-RD, which can involve the paranasal sinuses, other regions of the nasal cavity, the pterygopalatine fossa, the infraorbital nerve, and the nasopharynx. Head and neck CT was available in 27 patients, limiting the ability to systematically exclude additional sinonasal involvement. Although 12 (44.4%) of these 27 patients had concomitant sinusitis, none had additional mass-like lesions elsewhere in the sinonasal tract. These findings suggest that, at least among patients who underwent cross-sectional imaging, the inferior turbinate lesions were predominantly localized rather than part of multifocal mass-forming sinonasal disease.

Third, the diagnosis and classification of these nasal lesions remain challenging. The 2019 ACR/EULAR classification criteria were developed primarily for research classification and do not recognize the sinonasal tract as a typical site of IgG4-RD involvement (11). We therefore additionally applied the histopathologic and diagnostic framework of the 2011 comprehensive diagnostic criteria to the 18 patients who underwent biopsy (12). Of these, 9, 7, and 2 patients were classified as possible, probable, and definite IgG4-RD, respectively. Thus, even using the comprehensive diagnostic criteria, only 2 (11.1%) of the 18 biopsied patients fulfilled the criteria for definite IgG4-RD. Previous case reports and small case series have described sinonasal IgG4-RD diagnosed using comprehensive diagnostic criteria, including cases with isolated nasal turbinate involvement (23–26). Nevertheless, the distinctive localization of the lesions to the inferior turbinate, together with their clinicopathologic characteristics, suggests that the lesions observed in our cohort may not represent conventional systemic IgG4-RD. Further studies are needed to determine whether these lesions represent a localized IgG4-rich inflammatory response associated with chronic sinonasal inflammation, a single-organ manifestation within the spectrum of IgG4-RD, or a reproducible clinicopathologic phenotype distinct from conventional IgG4-RD.

In conclusion, lymphoid hyperplasia of the inferior turbinate characterized by IgG4-rich lymphoplasmacytic infiltration might represent a distinct benign inflammatory disease entity rather than sinonasal IgG4-related disease. Further studies are required to clarify its optimal management and long-term prognosis.

## Data Availability

The original contributions presented in the study are included in the article/supplementary material. Further inquiries can be directed to the corresponding author.
